# Comparison of automated and manual mRNA enrichment to automated rRNA depletion for whole-blood RNA-sequencing

**DOI:** 10.1038/s41598-025-32961-4

**Published:** 2025-12-30

**Authors:** Denis Awany, Helgard Claassen, Nadia Carstens, Simon C. Mendelsohn, Mzwandile Erasmus, Thomas J. Scriba, Al Leslie, Emily B. Wong

**Affiliations:** 1 South African Tuberculosis Vaccine Initiative, Institute of Infectious Disease and Molecular Medicine and Division of Immunology, Department of Pathology, University of Cape Town, Anzio Road, Observatory, Cape Town, 7935 South Africa; 2 Africa Health Research Institute (AHRI), Durban & Somkhele, South Africa; 3 South African Medical Research Council Genomics Platform, Cape Town, South Africa; 4 Division of Infection and Immunology, University College London, London, UK; 5 Division of Infectious Diseases, Department of Medicine, Heersink School of Medicine, University of Alabama Birmingham, Birmingham, AL USA

**Keywords:** RNA-sequencing, Messenger RNA enrichment, Ribosomal RNA depletion, Robotic library preparation, Manual library preparation, Differential gene expression, RNA sequencing, Tuberculosis

## Abstract

RNA sequencing libraries are typically processed before sequencing. We compared the performance of automated mRNA enrichment (autoE) and manual mRNA enrichment (manE) with globin depletion, and automated rRNA depletion (autoD) with globin depletion prior to sequencing. PAXgene tubes from 21 people living with HIV (PLWH) and 7 people without HIV were used, with RT-qPCR data from unprocessed RNA as an orthogonal comparator. Duplicate reads were higher for manE (58–61%) and autoE (59–70%) than autoD (29–33%). AutoE had the highest mapping to exonic regions (72–78% vs. 50–74% manE and 30–39% autoD). Globin transcripts were more abundant for autoD than autoE and manE, but remained low (median 10% vs. 0.1 and 1.3%). 94% of protein-coding genes were detected by all methods vs. 63–66% for other RNA-biotypes. All methods correlated strongly with qPCR data for highly expressed genes but not lowly expressed genes; concordance was lowest for autoE. When comparing PLWH with controls, manE and autoD identified more differentially expressed genes than autoE. While all methods exceeded the minimum threshold required for downstream analysis, autoD provided the most stable sequencing quality. In conclusion, manE and autoD yielded similar results with autoD providing advantages of robotic liquid handling for large-scale studies.

**Supplementary Information:**

The online version contains supplementary material available at 10.1038/s41598-025-32961-4.

## Introduction

Use of RNA sequencing (RNA-seq) has expanded significantly over the past decade, with applications including differential gene expression, isoform, and alternative splicing analysis, among others^1^. In translational research, transcriptomic profiling has become a powerful tool for interrogating and predicting various disease states, with promising applications ranging from disease diagnosis, to prediction of risk of progression to disease, and monitoring of response to treatment^2–10^. Prior to sequencing, processing of purified RNA is generally used to limit the sequencing of non-informative ribosomal RNA (rRNA), which constitutes more than 80% of total human RNA^11^. This can typically be achieved either by depletion of rRNA or enrichment of messenger RNA (mRNA) species using capture of polyadenylated RNA (poly-A enrichment). In addition, RNA purified from whole blood contains abundant haemoglobin RNAs, which are often depleted as they take up valuable sequencing depth and can interfere with detection of less abundant transcripts^12^. These approaches have advantages and disadvantages that have been explored by previous groups. However, these studies have caveats, including being limited by small sample size, use of non-human samples, samples limited to specific cell lines, analysis limited to specific RNA species, or the lack of data obtained from an orthogonal RNA quantification method to compare to the RNA-seq data^13–23^. Consequently, there is no clear consensus as to which method to use for human transcriptional studies. In addition, there is an increasing move to automate these steps for large scale sequencing projects that require consistency across hundreds or thousands of samples. The impact of automated extraction methods on downstream bioinformatic analyses is unknown. To address this knowledge gap, prior to conducting a large-scale study of whole blood transcriptional signatures of human tuberculosis (TB) that utilizes baseline samples from the population-based Vukuzazi cohort^24^, we conducted a comparative analysis of automated mRNA enrichment (autoE), and manual mRNA enrichment (manE), both with globin depletion, and automated rRNA depletion (autoD) with globin mRNA depletion on human whole-blood RNA samples for RNA sequencing. We assessed the quality of reads generated and alignment rates, capture efficiency for exonic regions, and specificity in profiling protein-coding and non-coding transcripts. To establish performance against a standard, we measured expression of a small subset of genes previously identified as having predictive value in the diagnosis of incipient TB disease in humans by real-time quantitative PCR (RT-qPCR), with no enrichment or depletion steps^25^. Finally, our dataset included people living with HIV (PLWH), which is known to perturb the whole blood transcriptome^26^, allowing us to determine the impact of RNA processing methods on downstream bioinformatic analysis methods including differential gene expression and pathway analysis.

## Results and discussion

**Fig. 1:**
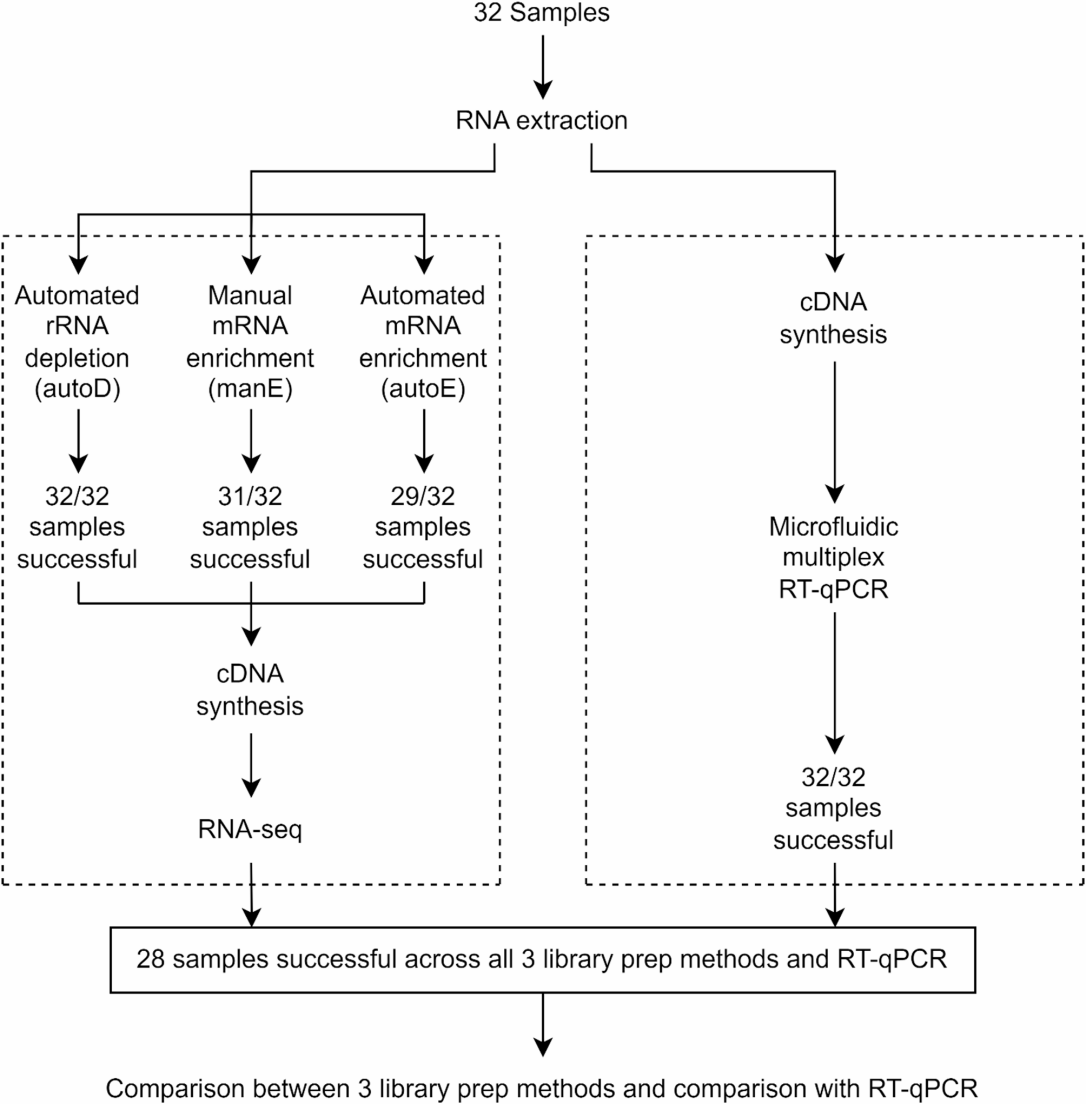
Study design and flow for the participant samples in the analysis.

The three RNA pre-processing methods were performed in parallel on sets of manually-extracted RNA samples collected from 32 participants, 21 of whom where PLWH with viral loads fully suppressed on antiretroviral therapy (ART). Of the samples from 32 participants, only 28 had samples successfully processed across all three methods, with failure being defined as RNA (post-processed) concentration < 3 (ng/µL) (Fig. 1). Notably, autoD was successful for all 32 samples, while 3% (1/32) and 9% (3/32) of samples failed manE and autoE, respectively, in our hands. All 32 samples were successfully processed for RT-qPCR. RNA libraries were sequenced on the MGI DNBSEQ-G400RS platform in paired-end mode to a length of 100 base pairs. After sequencing, reads were processed and analysed using the same analytical pipeline. There was no difference in sequencing depth across the three libraries (Kruskal-Wallis $\:p=0.11$); median sequencing depth was 60 million (IQR 57–62), 61 million (58–65), and 57 million (52–62) for autoD, manE, and autoE respectively.

### Read quality

**Fig. 2:**
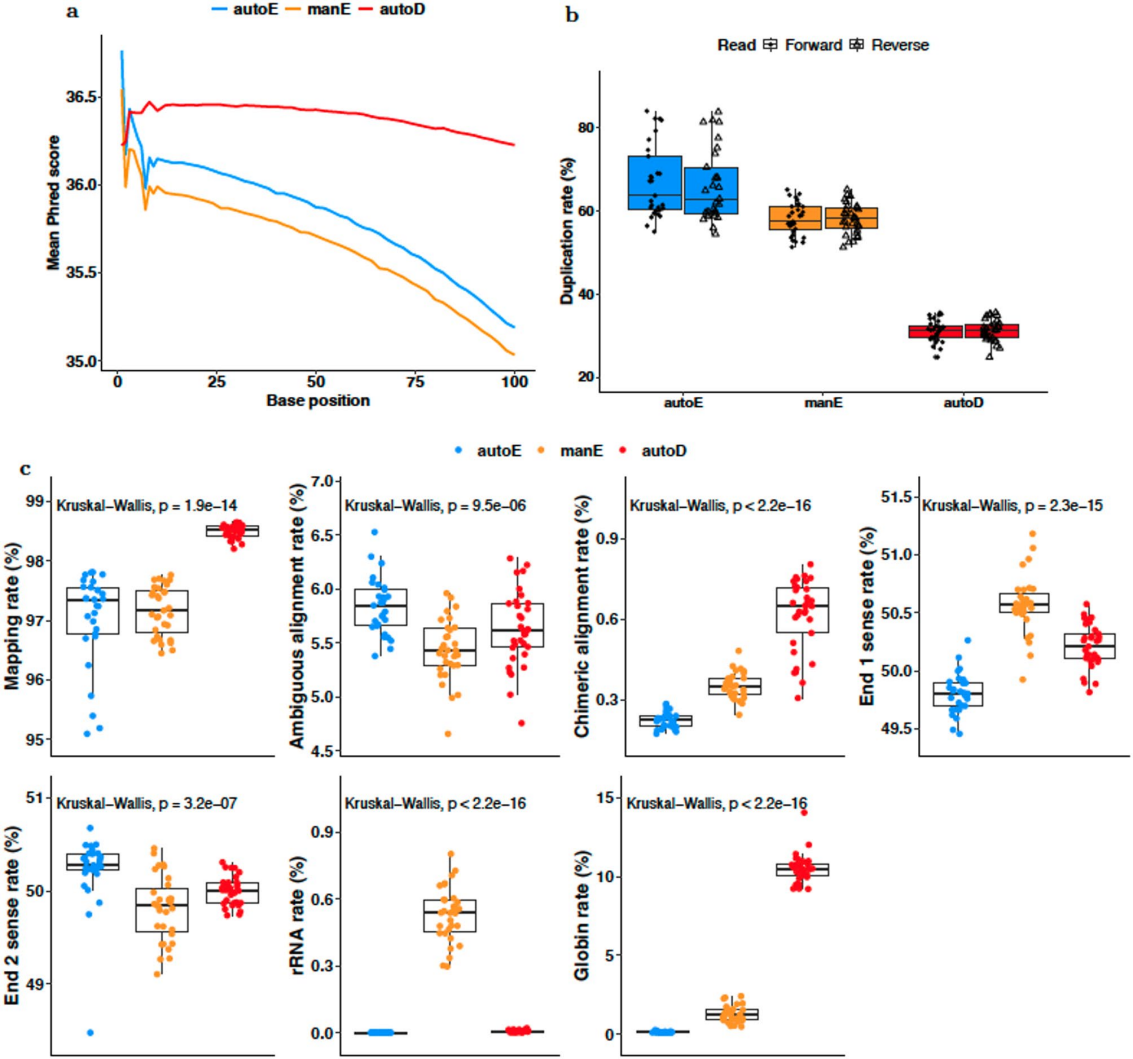
Alignment quality metrics (**a**) Per-base Phred quality scores for the three RNA-seq methods averaged across all samples for forward and reverse reads. (**b**) Rates of duplication reads. (**c**) Comparison of read mapping rates for reads from automated enrichment, manual enrichment, and automated depletion libraries. P-values based on Kruskal-Wallis statistics for the group comparison.

We first assessed the quality of sequence reads yielded by the three methods. While all methods resulted in high accuracy for per-base base calls, with mean Phred quality scores > 30, manE showed the largest decrease in Phred quality scores across read length, autoE exhibited moderate decreases, whereas autoD maintained consistently high quality with minimal drop along the length of the sequences (Fig. 2). Duplication rates were significantly different across the three methods (Kruskal-Wallis $\:p<{2\times\:10}^{-16}$). The median proportion of duplicate reads in the autoE library (63%; IQR 60–72%) was significantly higher than in the manE library (58%; IQR 55–61%), Mann-Whitney $\:p={4.7\times\:10}^{-9}$, and in the autoD library (31%; IQR 29–33%), Mann-Whitney $\:p={1.9\times\:10}^{-21}$. However, these rates are not concerning as reads with high proportion of highly duplicated reads (more than 10 copies per read) constituted a very small proportion (< 25%) of the libraries.

### Genome alignment statistics

The accuracy of read alignment directly determines the accuracy of transcript quantification from RNA-seq data. We therefore compared alignment metrics for reads from each of the three methods. Globally, there was a significant difference across the groups in the proportion of overall mapped reads (Kruskal-Wallis$\:\:\:p=1.9\times\:{10}^{-14}$) (Fig. 2). However, reads from all three methods had excellent rates of alignment, with ranges of 95.1–97.8%, 96.4–97.8%, and 98.2–98.6% for autoE, manE, and autoD respectively. Comparison of end 1 and end 2 sense rates revealed a significant difference in mean rate across the methods (Kruskal-Wallis $\:p<0.001$). However, despite the overall appreciable difference across the methods, the observed values remained tightly clustered within an acceptable range (49-51%), indicating that strand specificity was properly maintained in all methods and posed no practical concerns for the downstream analyses. Rates of ambiguous and chimeric alignments were all very low. Most notably, autoD exhibited a higher proportion of reads mapping to haemoglobin sequences than autoE and manE, consistent with less efficient globin depletion, although the NEBNext Globin & rRNA Depletion Kit did effectively reduce highly abundant haemoglobin transcripts.

**Fig. 3:**
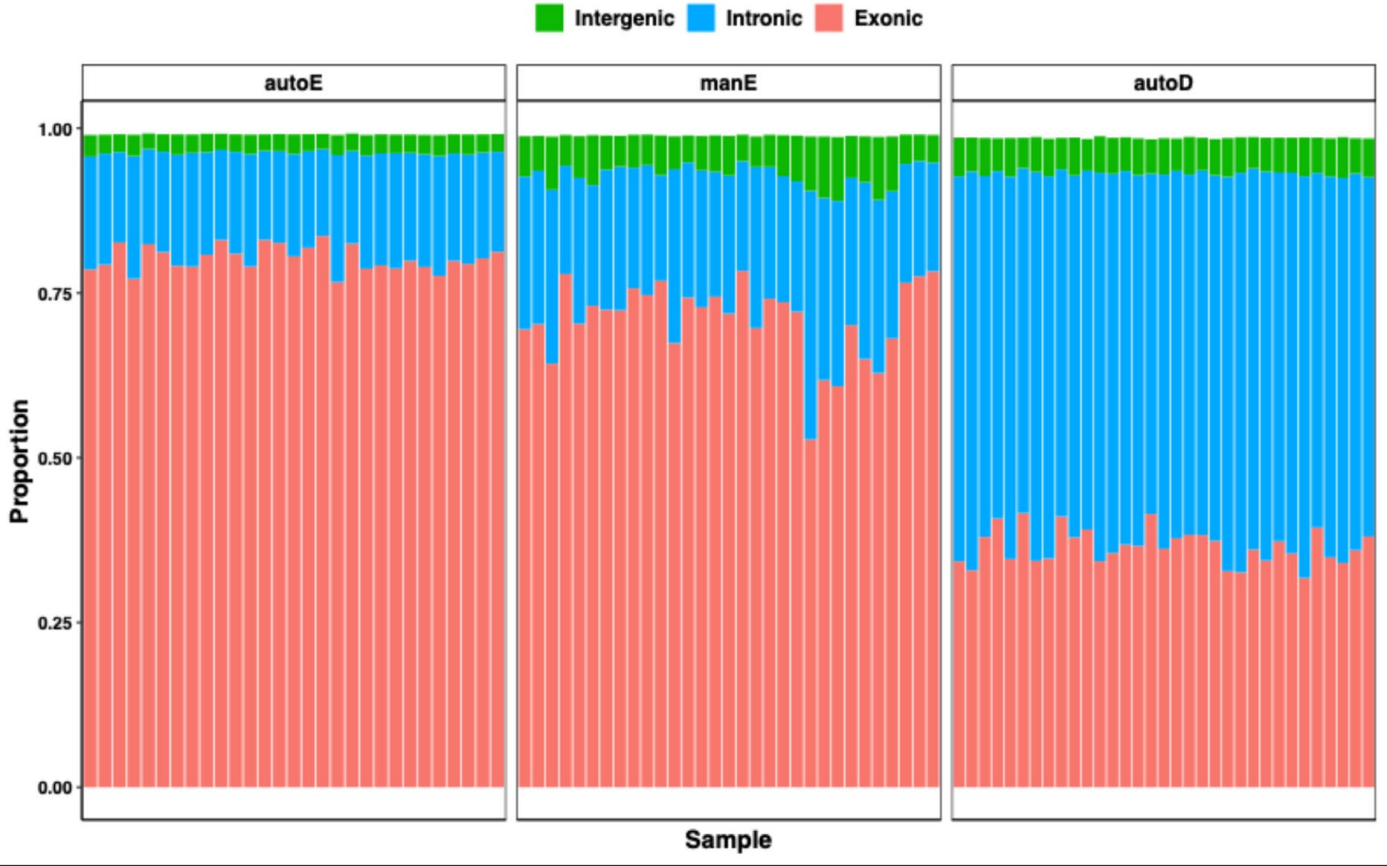
Rates of alignment to exonic, intronic, and intergenic regions for all samples. Rates expressed as a proportion of total reads. Each vertical bar corresponds to a sample.

Consistent with the different approaches, the protocols showed marked disparity in exonic and intronic read distribution; autoE resulted in the highest proportion of reads mapping to exonic regions (72–78%, mean 76%) followed by the manE library (50–74%, mean 68%), while the autoD libraries had the least mapping to exonic region (30–39%, mean 34%) (Fig. 3). This was expected and is in line with results from previous studies as only mature mRNAs are polyadenylated^15–21,23^. The proportions of intergenic reads for autoE, manE, and autoD were similar (mean 3%, 6%, and 5%, respectively). Although marginally higher for manE, only a tiny fraction of ribosomal RNAs were seen in the autoE (mean 0.001%), manE (mean 0.5%), and autoD (mean 0.007%) protocols. Previous studies have shown mixed results when comparing percentages mapped to rRNA between methods. Zhao et al. found similar percentages between rRNA depletion and mRNA selection protocols^14^, while Kumar et al. also had a lower percentage mapped to rRNA in their rRNA depletion protocol^15^ and Jaksik et al. reported more rRNA contamination in their rRNA depletion arm^21^. As high haemoglobin gene counts in RNA-seq libraries can take up a large proportion of reads, impacting the sensitivity of detection of non-haemoglobin protein-coding and non-coding RNAs, we estimated the mapping rate of total reads to globin sequences from ten protein-coding haemoglobin subunit genes and two pseudogenes from Ensembl (r91) (Supplementary Table S1). As expected, AutoE and manE libraries, both of which had a globin mRNA depletion step, had low levels of globin reads, averaging 0.1% and 1.3% of total reads, respectively. In contrast, autoD libraries, which also included globin depletion using the NEBNext Globin & rRNA Depletion Kit, had slightly higher globin read levels (rarely exceeding 10%), indicating that the depletion was effective but less efficient than for the enrichment-based approaches. Thus, while all three methods effectively prevent loss of sequencing depth due to rRNA contamination, relatively higher haemoglobin RNA abundance with autoD could impact downstream analysis.

### Gene detection

**Fig. 4:**
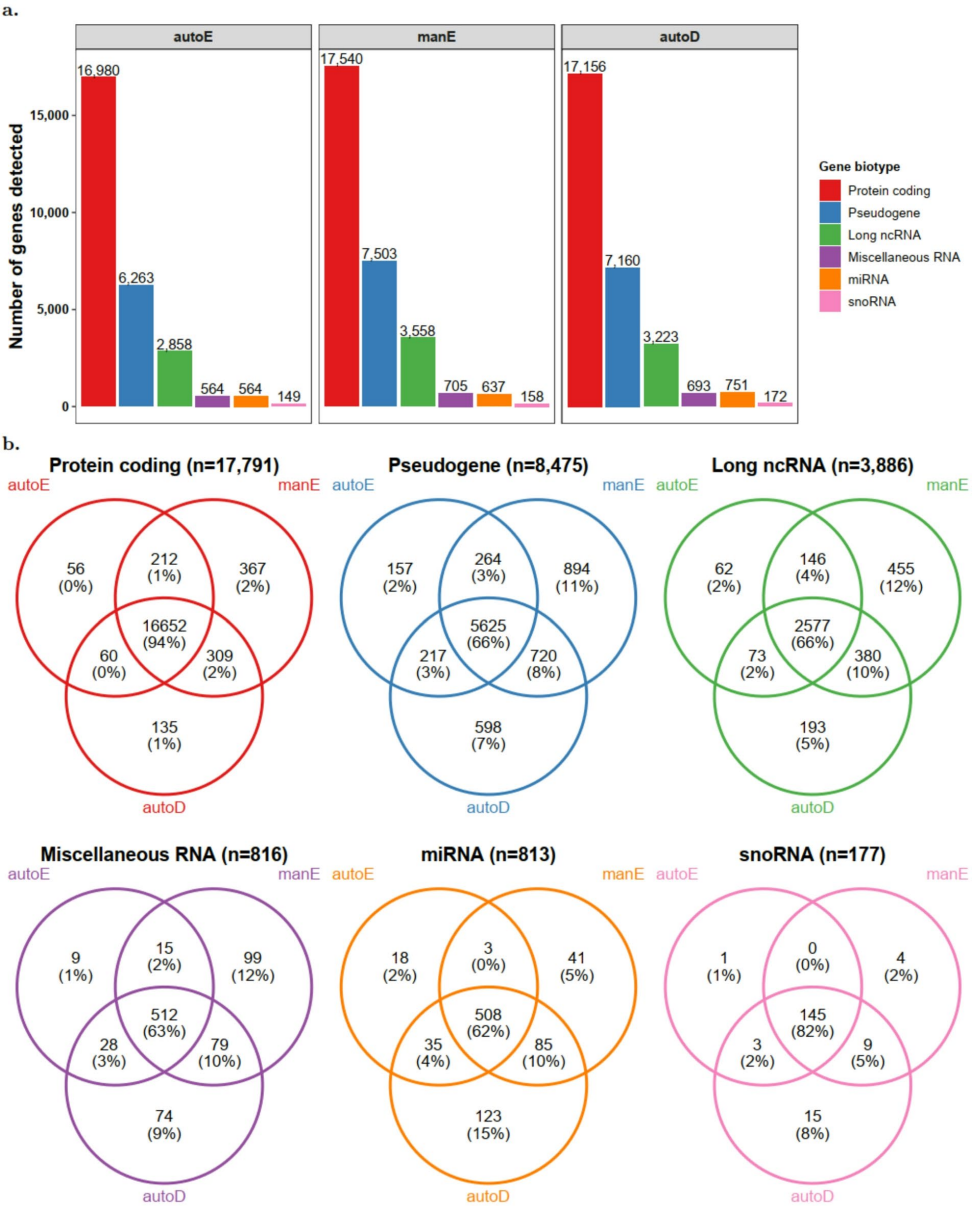
Number of genes detected by each automated mRNA enrichment (autoE), manual mRNA enrichment (manE), and automated rRNA depletion (autoD) methods. (**a**) Detected genes grouped by gene biotype, and (**b**) the total number of genes detected by all methods and overlap and differences between the genes detected. ncRNA, non-coding RNA. miRNA, microRNA. snoRNA, small nucleolar RNA.

Next, we evaluated the detection of the annotated genes and gene biotypes, including protein-coding genes, pseudogenes, long non-coding RNA (long ncRNA), miscellaneous RNA (miscRNA), microRNA (miRNA), and small nucleolar RNA (snoRNA) for each method. To account for differences in sequencing depth, gene counts were normalized to counts per million (CPM) prior to analysis. A gene was considered expressed in a sample if its CPM was greater than zero, and expressed genes in a sample were grouped into the six biotypes listed above. The number of genes detected per protocol varied by gene types (Fig. 4), although these differences were not statistically significant (Kruskal-Wallis $\:p=0.324$, $\:p=0.595$, $\:p=0.568$, $\:p=0.733$, $\:p=0.356$, and $\:p=0.350$ for protein-coding, pseudogene, long ncRNA, miscellaneous RNA, miRNA, and snoRNA, respectively). The highest concordance between the three methods was observed with protein-coding genes. Of all protein-coding genes detected, 94% were detected by all three protocols and 3% were detected in only one protocol. The few discordant genes (detected in only one of the protocols) were those with very low expression (Supplementary Fig. S1). ManE yielded the highest number of protein-coding genes, but this was not significantly different from the other methods (Kruskal-Wallis *p* = 0.324). Of all snoRNAs detected, 82% were common to all protocols. For less abundant RNA types, such as miRNA, miscellaneous RNA, long ncRNA, and pseudogenes, there were large discrepancies between the methods, with only 63–66% of elements being detected by all three protocols. These data are consistent with Zhao et al., who also reported greater discordance between mRNA enrichment and rRNA depletion for small RNA molecules, although it is not reported whether robotic (automated) or manual library preparation methods were utilized^17^. Previous comparisons have shown mixed results for long ncRNA detection. Jaksik et al. reported that mRNA enrichment and rRNA depletion methods performed equally well for long ncRNA detection^21^, while rRNA depletion outperformed mRNA selection in three studies^16,22,23^. However, these studies were based on tissues other than blood, or degraded and low-quantity samples, and did not report whether manual or robotic library preparations were used. Consistently across biotypes, manE and autoD detected genes that were not detectable by autoE, with autoD capturing more unique transcriptome features especially for small RNA molecules, as has also been previously described^14–17,19–22^.

### Correlation of gene expression measurements

**Fig. 5:**
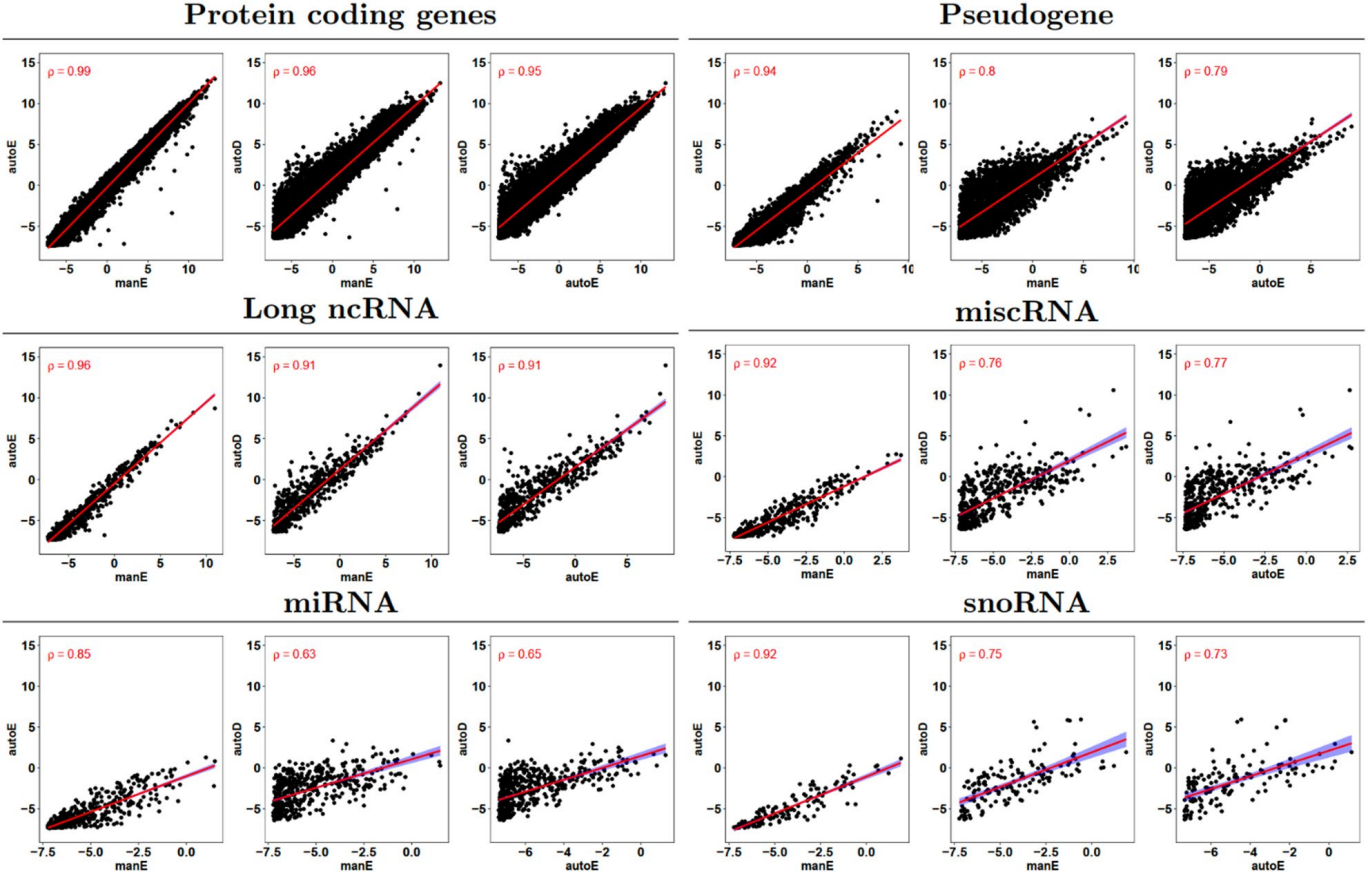
Correlation of gene expression measured by automated mRNA enrichment (autoE), manual mRNA enrichment (manE), and automated rRNA depletion (autoD) methods, based on all 28 common samples. The x and y axes correspond to log2 (CPM)-normalized expression values and the Spearman correlation coefficient ($\:\rho\:$) is shown in the top-left corner of each plot. ncRNA, non-coding RNA. miscRNA, miscellaneous RNA. miRNA, microRNA. snoRNA, small nucleolar RNA.

We next examined how the methods differ in their gene expression profiles. Gene expression levels were compared in a paired analysis on the 28 samples profiled by all three protocols. To permit direct comparison, we restricted this correlation analysis to the list of genes detected in all three protocols. ManE and autoE showed the highest correlation across RNA biotypes (Spearman rho 0.85–0.99). The correlation of autoD with manE and autoE methods was high for protein-coding and low for miRNA (Spearman $\:\rho\:=0.96$ for protein-coding compared to $\:\rho\:=0.63$ for miRNA, and $\:\rho\:=0.95$ for protein-coding compared to $\:\rho\:=0.65$ for miRNA, respectively—Fig. 5). Overall, the highest correlation was observed with protein-coding genes, followed by long ncRNAs, and other small and less abundant RNAs. Previous comparisons have also reported high levels of correlation between methods^15,20–22^. It is also evident that autoE exhibits higher degree of inter-sample variability compared to manE and autoD methods (Fig. 5, Supplementary Fig. S2).

### Comparison of RNA-seq and TaqMan RT-qPCR measurements

The above analyses lack an independent comparator group to evaluate the effect of RNA processing on gene expression. Therefore, we measured the expression level of 21 genes in unprocessed RNA from the same individuals using a highly optimised microfluidic RT-qPCR with TaqMan primer/probe assays. The genes in question are largely interferon response genes that have been shown to be differentially expressed between TB patients and healthy controls, and five genes that have been suggested as being potentially predictive of protection from TB disease^9,25,27–30^. Importantly for this analysis, this gene-set includes both highly and lowly expressed genes, allowing us to determine the impact of the three methods across a range of gene expression.

**Fig. 6:**
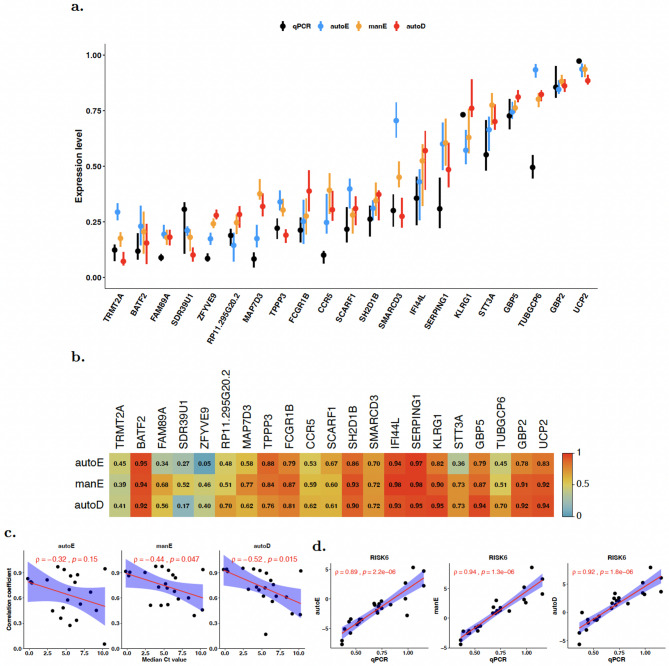
Comparison of measurement of expression for selected genes. (**a**) Normalized expression levels (scaled to 0–1 range) of genes from each platform. Point and vertical line correspond to the median and interquartile range of the expression value, and genes have been ordered from left to right by the median expression level. (**b**) Pearson correlation coefficients between gene expression measured by microfluidic qPCR and gene expression measured by automated mRNA enrichment (autoE), manual mRNA enrichment (manE), and automated rRNA depletion (autoD) methods. (**c**) Pearson correlation between the correlation coefficients and the median Ct values; each dot corresponds to a gene and the scatter plot is obtained by plotting the correlation coefficient of the gene (between qPCR and RNA-seq measurements) and the median Ct value of the gene across samples. (**d**) Correlation between signature score obtained from RT-qPCR and RNA-seq (by either autoE, manE or autoD method) for RISK6 gene signature.

We found that the correlation between read counts for rRNA depletion and mRNA enrichment methods, and RT-qPCR normalised cycle threshold, varied widely across genes (Spearman rho range 0.05–0.98; Fig. 6). Notably, the correlations are generally stronger for highly expressed genes and weaker for the less abundant genes. Overall, however, correlation with qPCR data was weakest for autoE (*p* = 0.02 by fishers exact; Fig. 6B). Indeed, there was no statistically significant correlation between autoE and qPCR data (Fig. 6C). Previous studies have compared RNAseq data to an orthogonal method. Similar to these data, Chao et al. found that rRNA depletion and mRNA enrichment were similar to their qPCR data^23^. However, Schuierer et al. and Kumar et al. reported that their mRNA enrichment protocol correlated better with their qPCR data than their rRNA depletion protocol did^15,16^. Nevertheless, while individual genes measured using the RNA-seq and qPCR methods demonstrate variability in correlation—with some genes showing relatively weak concordance—the strength of the correlation increases significantly when analysed at the level of gene signatures. Gene signatures aggregate expression patterns across a set of related genes, providing a more robust and biologically meaningful measure of activity. This approach reduces the noise inherent in individual gene measurements and highlights the collective behaviour of gene groups, which often aligns more closely across methodologies (Fig. 6D).

This strong correlation at the gene signature level suggests that the three RNA-seq methods effectively capture the underlying biological processes, even if individual gene-level variability exists. This is particularly valuable in contexts such as pathway analysis, where the combined expression of multiple genes reflects the activation of specific biological pathways, enabling more reliable comparisons between datasets generated by different methods. Such findings also underscore the importance of focusing on sets of genes rather than isolated genes when aiming to draw broader biological insights.

### Differential gene expression

**Fig. 7:**
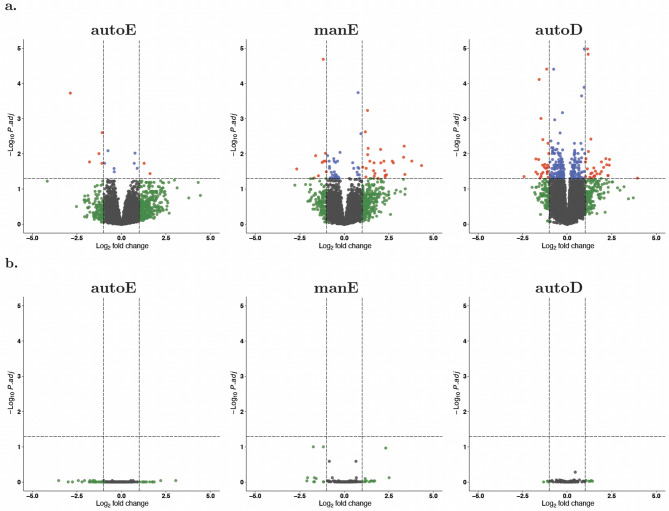
Volcano plot for differentially expressed protein-coding genes (**a**) and small non-coding RNA (snoRNA) (**b**) in the analysis of HIV-positive vs. HIV-negative samples. Horizontal and vertical dotted lines indicating significance thresholds of false discovery rate (FDR)-adjusted *p* < 0.05 and $\:\left|{log}_{2}\right|FC>1$, respectively. manE, manual mRNA enrichment. autoE, automated mRNA enrichment. autoD, automated rRNA depletion.

Next, we sought to compare the impact of these RNA processing methods on downstream bioinformatic analysis by quantifying differential gene expression between PLWH and HIV uninfected participants. It is important to note that all PLWH were on ART and had viral loads of less than 200 copies/ml. This reduces the transcriptional response detectable in whole blood, but persistent differential gene expression is reported even in long-term treated HIV^26^. Differential expression analysis between PLWH (*n* = 21) and their HIV negative counterparts (*n* = 7) identified a total of 39, 7, and 55 differentially expressed genes (DEGs) ($\:q<0.05$ and $\:\left|{log}_{2}FC\right|>1$) with the manE, autoE, and autoD protocols respectively (Fig. 7, Supplementary Table S3). Although the effect sizes for the detected DEGs are appreciably low to infer a reliably robust difference, it is striking that, in line with the lack of correlation with qPCR data, autoE is associated with the lowest number of DEGs. In addition, there was a surprising lack of overlap of DEGs, even between manE and autoD (~ 23%; Supplementary Fig. S4). As with the genes detected by each of the RNA-seq methods, most of the genes that were differentially expressed in only one RNA-seq method were those with comparatively low gene expression values (Supplementary Fig. S3). Kumar et al. and Chao et al. found that their mRNA enrichment protocols detected slightly more DEGs^15,23^, which was not observed here. It is important to note, however, the relatively large imbalance in the case/control ratio for this HIV group DEG analysis, which can compromise the statistical power to detect true associations. For the other biotypes (pseudogene, miscellaneous RNA, snoRNA, long ncRNA, and miRNA), there was no difference in the number of gene elements.

### Pathway analysis of gene expression

**Fig. 8:**
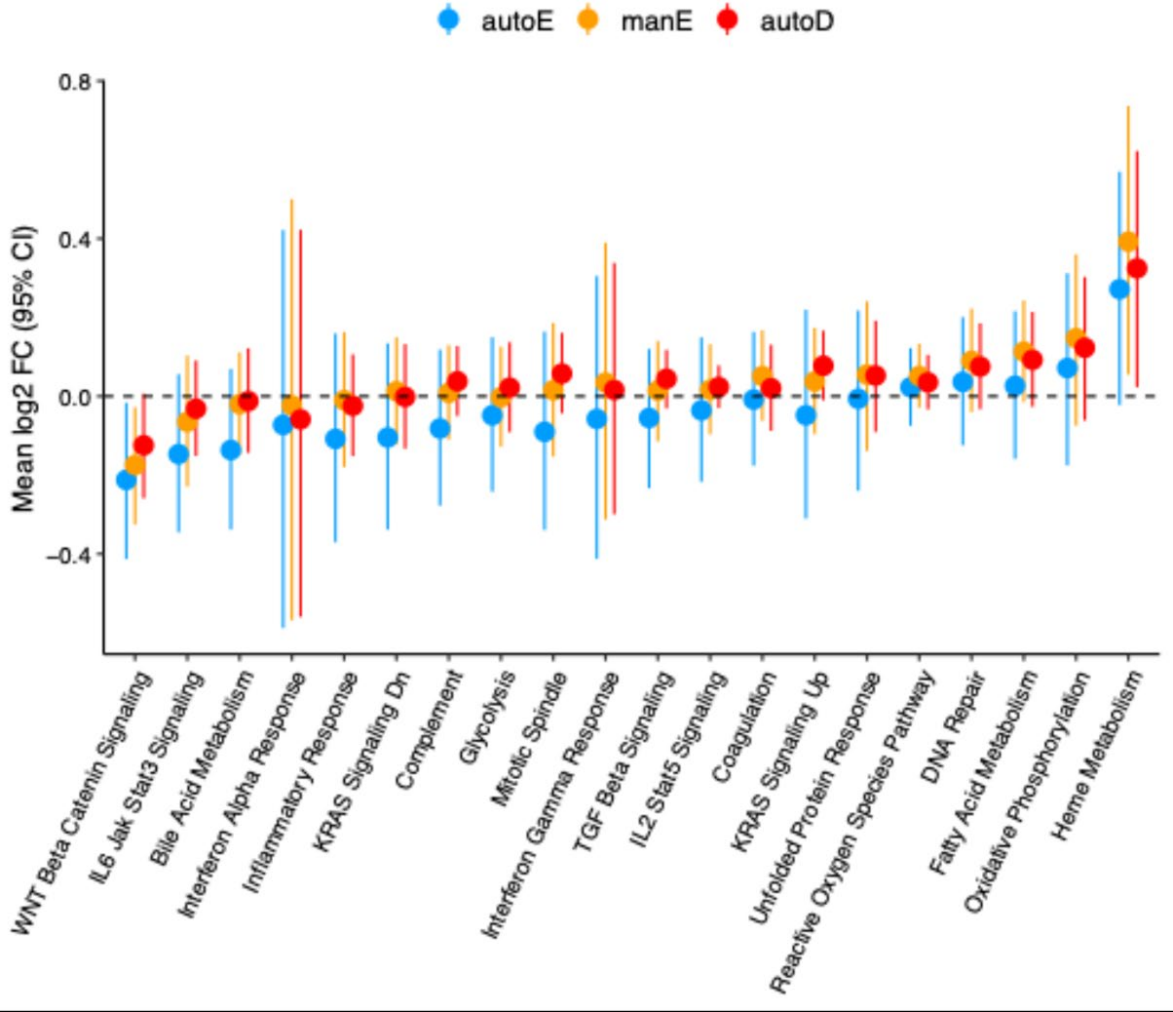
Pathway analysis of samples automatic mRNA enrichment (autoE), manual mRNA enrichment (manE), and automatic rRNA depletion (autoD) protocols in analysis of HIV-positive relative to HIV-negative samples. Fold change depicts the degree of modulation of the pathway in HIV-positive, relative to HIV-negative, samples. Shown are the mean and the 95% confidence intervals for the perturbation of the genes in the pathway. Analysis is based on expressed protein-coding genes in each protocol.

Finally, having identified only a small number of significantly DEGs, we tested whether a more sensitive gene pathway enrichment analysis would identify bigger differences between RNA processing methods. We used Gene Set Enrichment Analysis (GSEA) to compute enrichment scores of individual samples, and employed QuSAGE^31^ to test for differential expression of gene set pathways between case and control samples, utilizing MSigDB (v7.2)^32^ hallmark gene sets. Although the confidence intervals are highly overlapping, it is striking that, in line with previous analyses, manE and autoD are highly similar, while autoE tends to separate slightly (Fig. 8). A similar pathway analysis by Kumar et al. also showed concordance in pathway analysis between the two methods, although their rRNA depletion protocol produced slightly more significant enrichments^15^.

## Conclusions

In this study, we evaluated the concordance of transcript measurements in whole-blood derived total RNA samples processed in parallel by automated mRNA enrichment (autoE) and manual mRNA enrichment (manE) with globin mRNA depletion, and automated rRNA depletion (autoD) with globin depletion prior to sequencing. While previous investigations have often focused on the choice of enrichment/depletion methods, our head-to-head comparison across manual and automated platforms, benchmarked with RT-qPCR measurement, offers a more integrated and practical assessment and includes two automated methods that utilise robotic liquid handling making them advantages for large-scale studies. Overall, concordance for the three methods was high for protein-coding genes, but lower for the less abundant RNA species, with lower levels of detection by autoE accounting for this difference. ManE captures a slightly higher number of unique biotypes, although the differences between methods were not significant. Concordance with gene expression measured in unprocessed RNA by qPCR using an optimised 21 gene panel was high in all three methods for highly expressed genes, but less good for lowly expressed genes. AutoE processed RNA tended to correlate least well at the individual gene level and, overall, there was no significant correlation. In addition, manE and autoD identified a greater number of DEGs and were more concordant in terms of pathway analysis than the autoE RNA processing approach used here. In addition, although globin transcripts accounted for approximately 10% of transcripts using autoD, we did not detect a negative downstream impact. The fact that the rRNA and globin depletion steps are combined in the autoD method can also be advantageous since it reduces the number of library handling steps.

We conclude from these results that both manual mRNA enrichment (manE) and automated rRNA depletion (autoD) approaches yielded high quality RNA sequence data and were generally similar for the investigation of protein-coding and highly expressed genes and that either approach is suitable for studies based on protein-coding genes. However, the automated mRNA enrichment did not perform as well in our hands. Notably, manE and autoD also captured slightly higher numbers of unique transcriptome biotypes. Since the automated rRNA depletion (autoD) protocol utilizes robotic liquid handling and results in high quality RNAseq data, it will likely be useful for large-scale transcriptional studies.

## Materials and methods

### Study design, RNA sample collection and extraction

Participants are part of the ongoing SIPRO-TB (SIgnatures of PROtection-TB) study, which follows people who took part in the “Vukuzazi” multi-disease community-based screening program to determine if candidate whole blood transcriptomic signatures associated with protection against TB in animal models can predict human TB disease outcomes over six years of follow-up. The Vukuzazi study (which means “wake up and know yourself” in isiZulu) enrolled 18,041 participants 15 years and older from May 2018 to March 2020^24,33^. Participants were screened for HIV, TB, elevated blood glucose, and elevated blood pressure. The detailed methodology has been described in the cohort profile by Gunda et al.^24^, but the relevant study activities will be summarized here. HIV screening included blood collection for enzyme immunoassay testing (Genscreen Ultra HIV Ag-Ab enzyme immunoassay, Bio-Rad, CA, USA). If positive, a reflex HIV-1 RNA viral load (RealTime HIV-1 Viral Load, Abbott, IL, USA) and CD4 count was performed. TB screening included a digital chest x-ray and sputum collection for Xpert MTB/RIF Ultra testing (Cepheid, CA, USA) and liquid mycobacterial culture (BACTEC MGIT 960 System, Becton Dickinson, Berkshire, UK). Digital chest x-rays were assessed by computer-aided detection for TB (CAD4TB, Delft Imaging, Hertogenbosch, The Netherlands) software and a specialist radiologist. Venous blood was collected in PAXgene RNA tubes (PreAnalytiX, Hombrechtikon, Switzerland) and cryopreserved.

SIPRO-TB cases are participants who had no microbiological or radiological evidence of TB at baseline, but then went on to develop incident TB over the subsequent six years. Incident TB episodes are identified through the Africa Health Research Institute (AHRI) health surveillance program and linkage to the Department of Health (DoH) Tier.net and the National Health Laboratory Service (NHLS) databases. Each case is matched to two controls, who remained disease-free, by age, sex, HIV status, and geospatial proximity. Participants complete a follow-up visit to ensure correct classification. Follow-up visits consist of a TB questionnaire, chest x-ray, sputum collection for Xpert MTB/RIF Ultra test and liquid mycobacterial culture (BACTEC MGIT 960 System), and blood collection for HIV viral load and CD4 count. Cases and controls who are living with HIV are excluded if their CD4 count was < 200 cells/mm^3^ or their HIV viral load was > 200 copies/ml at baseline (Vukuzazi). Cases are also excluded if they meet these exclusion criteria at follow-up.

Baseline (Vukuzazi) PAXgene samples were thawed and RNA was extracted using the PAXgene Blood RNA Kit (Qiagen, Hilden, Germany) according to manufacturers’ instructions, including DNase treatment. RNA integrity was assessed using the TapeStation RNA ScreenTape and Reagents (Agilent, CA, USA) with all samples having an RNA integrity number (RIN) above 7. RNA purity was assessed using a Multiskan SkyHigh spectrophotometer (Thermo Fisher Scientific, MA, USA) and RNA yield was quantified using the Qubit RNA Board Range assay kit (Thermo Fisher Scientific, MA, USA).

### Library construction and RNA-seq

The same 32 RNA samples were processed in parallel according to each of the three enrichment strategies to generate the three sample sets prior to library preparation (autoD, manE, and autoE). The manE sample set was prepared manually starting with 1000ng RNA. Globin depletion was performed using the GLOBINclear Human Kit (Invitrogen, MA, USA) followed by mRNA purification using the Dynabeads mRNA DIRECT Kit (Invitrogen, MA, USA). The autoD and autoE sample sets were prepared on the MGISP-960 High-throughput Automated Sample Preparation System starting with 1000ng RNA. The autoD sample set was processed using the NEBNext Globin & rRNA Depletion (New England Biolabs, MA, USA) kit only. The autoE sample set was enriched for poly(A) mRNA using the NEBNext Poly(A) mRNA Magnetic Isolation Module (New England Biolabs, MA, USA), followed by globin depletion using the NEBNext Globin & rRNA Depletion Kit (New England Biolabs, MA, USA).

All samples were then prepared for sequencing using MGIeasy RNA Library Prep kit (MGI Tech, Shenzhen, China). This protocol converted RNA to double-stranded cDNA (ds-cDNA). The ds-cDNA were subjected to heat, denatured, and circularised to generate single stranded circular DNA. This was followed by rolling circle replication to create DNA nanoballs (DNB) and these were sequenced on the MGI DNBSEQ-G400RS to generate paired-end sequences of 100 base pairs (bp) using the DNBSEQ-G400RS High-throughput Sequencing Set (FCL PE100) (MGI Tech, Shenzhen, China) according to manufacturer’s instructions.

### Microfluidic RT-qPCR

Genes from six published TB transcriptomic signatures^9,27–30^ and three reference genes were translated to RT-qPCR and measured as previously described^25^. Briefly, RNA samples were thawed, cDNA synthesised with EpiScript reverse transcriptase (Lucigen, Middleton, WI, USA), and genes of interest pre-amplified using pools of TaqMan gene expression primer-probe assays (Supplementary Table S2; Thermo Fisher Scientific, MA, USA). Gene expression (raw cycle threshold, Ct) was subsequently quantified by microfluidic RT-qPCR using Standard BioTools (formerly Fluidigm, CA, USA) 192.24 (192 samples multiplexed with 24 primer-probe assays) Gene Expression chips on a BioMark HD instrument (Standard BioTools). Quality control filters and gene expression normalisation were performed using a pre-defined R script (Supplementary Methods). Normalised gene expression (delta Ct) was calculated as the raw Ct value of the gene of interest minus the geometric mean of the raw Ct values for the three reference primer-probes.

### Reads quality control, alignment, and counting

Paired-end sequences of length 100 bp were quality-checked using FastQC (v0.12.0)^34^ and MultiQC (v1.2.1)^35^. Cutadapt (v4.8)^36^ was used to check and remove any existing adapter sequences. Quality-filtered reads were aligned to the human genome (GRCh38.p13), using HISAT2 (v2.2.1)^37^ with default parameters across all comparisons. Unaligned reads were discarded. Post-alignment QC metrics were obtained using RNA-SeQC (v2.4.2)^38^. For calculation of the proportion of reads mapping to globin reads, we utilized sequences from 12 hgRNAs from Ensembl annotation, obtained from Harrington et al.^12^ (Supplementary Table S1).

Expression of transcripts was quantified using *featureCounts* (v2.0.0)^39^, generating raw counts of transcript abundance. Normalized expression was obtained by applying *voom* transformation^40^ to the raw counts to obtain expression data on $\:{log}_{2}$-scale.

### Differential expression of genes and pathway regulation

Analysis to identify differentially-expressed (DE) genes was performed on raw counts using DESeq2, fitting the Wald significance test. For each method, namely autoE, manE, or autoD RNA-seq, differential expression was tested by comparing baseline samples for PLWH (*n* = 21) vs. HIV negative participants (*n* = 7), on samples profiled by all the three RNA-seq methods. For analyses by gene biotype, biotype classifications were obtained from Ensembl (EnsDb.Hsapiens.v75; v2.99.0). Statistical significance for a DE gene was defined as false discovery rate (FDR)-adjusted $\:p-value<0.05$ and absolute $\:{log}_{2}FC>1$. We used Gene Set Enrichment Analysis (GSEA) to find enrichment in MSigDB (v7.2)^32^ hallmark gene sets, and utilized QuSAGE^31^ to obtain fold change in enrichment scores. Due to the relatively small numbers of this pilot study, which was designed to inform which library preparation method to use for the SIPRO-TB study, we did not compare baseline sequencing data of SIPRO-TB cases to controls in any of the analyses.

## Supplementary Information

Below is the link to the electronic supplementary material.

Supplementary Material 1

Supplementary Material 2

Supplementary Material 3

Supplementary Material 4

## Data Availability

The raw and processed RNA-seq data analysed is available at Gene Expression Omnibus (GEO; accession number PRJNA1364186).
