# Supplementary material for: Comparison of automated and manual mRNA enrichment to automated rRNA depletion for whole-blood RNA-sequencing
Source: Sci Rep. 2025 Dec 30;16:3156. doi: 10.1038/s41598-025-32961-4 (PMC12830761; doi:10.1038/s41598-025-32961-4)
Supplement: Supplementary file 1 — Supplementary Material 1 [file 41598_2025_32961_MOESM1_ESM.docx]

**Supplementary Table S1. Human haemoglobin gene family.**

| **Ensemble ID** | **Gene name** | **Gene biotype** | **Gene description** |
| --- | --- | --- | --- |
| ENSG00000206172 | HBA1 | protein_coding | haemoglobin subunit alpha 1 [Source:HGNC Symbol;Acc:HGNC:4823] |
| ENSG00000188536 | HBA2 | protein_coding | haemoglobin subunit alpha 2 [Source:HGNC Symbol;Acc:HGNC:4824] |
| ENSG00000244734 | HBB | protein_coding | haemoglobin subunit beta [Source:HGNC Symbol;Acc:HGNC:4827] |
| ENSG00000229988 | HBBP1 | transcribed_unprocessed_pseudogene | haemoglobin subunit beta pseudogene 1 [Source:HGNC Symbol;Acc:HGNC:4828] |
| ENSG00000223609 | HBD | protein_coding | haemoglobin subunit delta [Source:HGNC Symbol;Acc:HGNC:4829] |
| ENSG00000213931 | HBE1 | protein_coding | haemoglobin subunit epsilon 1 [Source:HGNC Symbol;Acc:HGNC:4830] |
| ENSG00000213934 | HBG1 | protein_coding | haemoglobin subunit gamma 1 [Source:HGNC Symbol;Acc:HGNC:4831] |
| ENSG00000196565 | HBG2 | protein_coding | haemoglobin subunit gamma 2 [Source:HGNC Symbol;Acc:HGNC:4832] |
| ENSG00000206177 | HBM | protein_coding | haemoglobin subunit mu [Source:HGNC Symbol;Acc:HGNC:4826] |
| ENSG00000086506 | HBQ1 | protein_coding | haemoglobin subunit theta 1 [Source:HGNC Symbol;Acc:HGNC:4833] |
| ENSG00000130656 | HBZ | protein_coding | haemoglobin subunit zeta [Source:HGNC Symbol;Acc:HGNC:4835] |
| ENSG00000206178 | HBZP1 | unprocessed_pseudogene | haemoglobin subunit zeta pseudogene 1 [Source:HGNC Symbol;Acc:HGNC:4836] |

Supplementary Table S2. TaqMan PCR primer-probe panel for 192.24 gene expression chip.

| **Gene Symbol** | **Gene Name** | **TaqMan Assay ID** | **Reference probe** | **Herberg2** | **Duffy5** | **Penn-Nicholson6** | **Roe1** | **Roe3** | **Thompson5** |
| --- | --- | --- | --- | --- | --- | --- | --- | --- | --- |
| ACTR3 | ARP3 actin related protein 3 homolog | Hs01029159_g1 | X | X | X |  | X | X |  |
| TMBIM6 | Transmembrane BAX inhibitor motif containing 6 | Hs00162661_m1 | X | X | X |  | X | X |  |
| USF2 | Upstream transcription factor 2, c-fos interacting | Hs01100994_g1 | X | X | X |  | X | X |  |
| BATF2 | Basic leucine zipper ATF-like transcription factor 2 | Hs00912737_m1 |  |  |  |  | X | X |  |
| CCR5 | C-C motif chemokine receptor 5 (gene/pseudogene) | Hs99999149_s1 |  |  | X |  |  |  |  |
| FAM89A | Family with sequence similarity 89 member A | CUSTOM_ARZTE3U* |  | X |  |  |  |  |  |
| FCGR1B | Fc fragment of IgG receptor Ib | Hs02341825_m1 |  |  |  | X |  |  |  |
| GBP2 | Guanylate binding protein 2 | Hs00894846_g1 |  |  |  | X |  |  |  |
| GBP5 | Guanylate binding protein 5 | Hs00369472_m1 |  |  |  |  |  | X |  |
| IFI44L | Interferon induced protein 44 like | Hs00915292_m1 |  | X |  |  |  |  |  |
| KLRG1 | Killer cell lectin like receptor G1 | Hs00929964_m1 |  |  | X |  |  |  |  |
| MAP7D3 | MAP7 domain containing 3 | Hs00226257_m1 |  |  |  |  |  |  | X |
| RP11-295G20.2 | RP11-295G20.2 | Hs01373568_m1 |  |  |  |  |  |  | X |
| SCARF1 | Scavenger receptor class F member 1 | Hs01092483_m1 |  |  |  |  |  | X |  |
| SDR39U1 | Short chain dehydrogenase/reductase family 39U member 1 | Hs01016970_g1 |  |  |  | X |  |  |  |
| SERPING1 | Serpin family G member 1 | Hs00934329_m1 |  |  |  | X |  |  |  |
| SH2D1B | SH2 domain containing 1B | Hs01114628_m1 |  |  | X |  |  |  |  |
| SMARCD3 | SWI/SNF related, matrix associated, actin dependent regulator of chromatin, subfamily d, member 3 | Hs01088251_g1 |  |  |  |  |  |  | X |
| STT3A | STT3A, catalytic subunit of the oligosaccharyltransferase complex | Hs00967491_m1 |  |  |  |  |  |  | X |
| TPPP3 | Tubulin polymerization promoting protein family member 3 | Hs00372228_g1 |  |  | X |  |  |  |  |
| TRMT2A | tRNA methyltransferase 2 homolog A | Hs01000041_g1 |  |  |  | X |  |  |  |
| TUBGCP6 | Tubulin gamma complex associated protein 6 | Hs00363509_g1 |  |  |  | X |  |  |  |
| UCP2 | Uncoupling protein 2 | Hs01075224_g1 |  |  |  |  |  |  | X |
| ZFYVE9 | Upstream transcription factor 2, c-fos interacting | Hs01024382_m1 |  |  | X |  |  |  |  |

Signatures are named by first author and number of transcripts included in the model (e.g. Author11). Numbers in brackets indicate the original number of transcripts in the published model.

* Custom designed primer-probe assays; available on request to corresponding author.
