# Supplementary material for: Comparison of automated and manual mRNA enrichment to automated rRNA depletion for whole-blood RNA-sequencing
Source: Sci Rep. 2025 Dec 30;16:3156. doi: 10.1038/s41598-025-32961-4 (PMC12830761; doi:10.1038/s41598-025-32961-4)
Supplement: Supplementary file 2 — Supplementary Material 2 [file 41598_2025_32961_MOESM2_ESM.docx]

**Supplementary Methods**

**RT-qPCR data quality control**

Standard BioTools 96.96 gene expression data were analysed using a pre-defined R script with quality control filters that assessed the integrity and reproducibility of each chip. The following parameters were applied for extracting Ct values: Linear (Derivative) baseline correction, Quality Threshold of 0.3, and Auto (Global) for Ct Threshold Method using Standard BioTools Biomark software version 4.5.2. No-template (water) and internal positive control samples were run on each chip. Chips with marked deviation (> 5 standard deviations, Spearman correlation < 0.98, or concordance correlation coefficient < 0.95) in the internal positive control sample primer-probe assay raw Ct values or Penn-Nicholson6 (RISK6) signature score versus 11 historical runs, with amplification (raw Ct < 35 cycles) for any assay in the no-template control, or with more than 10% failed primer-probes, were repeated. Individual samples with more than 20% failed primer-probe reactions were classified as failed and no signature scores were computed. If less than 20% of primer-probe reactions failed for an individual sample, signature scores were computed where possible and signatures with missing primer-probe raw Ct values were deemed failed for that sample. Samples and primer-probe assays were run in singlet. Failed signature results for individual samples were assumed to follow a random distribution, thus not repeated, and excluded from analysis.
