# Supplementary material for: Comparison of automated and manual mRNA enrichment to automated rRNA depletion for whole-blood RNA-sequencing
Source: Sci Rep. 2025 Dec 30;16:3156. doi: 10.1038/s41598-025-32961-4 (PMC12830761; doi:10.1038/s41598-025-32961-4)
Supplement: Supplementary file 4 — Supplementary Material 4 [file 41598_2025_32961_MOESM4_ESM.docx]

**Supplementary Figure S1. Mean expression of genes detected by the three RNA-seq protocols.** Mean expression obtained by averaging the expression of a gene across all samples and assays. Genes detected in only one of the three protocols are classified as “1” and those detected in two or more as classified as “> 1”.

**Supplementary Figure S2. PCA of gene expression measurement from the three RNA-seq protocols.** PCA of log2 (CPM)-normalized gene expression measured by automatic mRNA enrichment (“autoEnrich”), manual mRNA enrichment (“manEnrich”), and automatic rRNA depletion (“autoDeplet”). Slightly higher variability was observed with automatic mRNA enrichment, whereas samples from the automatic rRNA depletion exhibited the least degree of variability.

**Supplementary Figure S3. Mean expression of genes differentially expressed in the three RNA-seq protocols.** Mean expression obtained by averaging the expression of a gene across all samples and assays. Genes differentially expressed in only one of the three protocols are classified as “1” and those differentially expressed in two or more as classified as “> 1”.

**Supplementary Figure S4. Intersection of the genes detected as significantly differentially expressed.** Venn diagram for the intersection of the genes differentially expressed in the analysis of HIV-positive vs HIV-negative samples using each gene biotype. Only biotypes with at least one differentially expressed gene is shown here.
